# Clinical and Molecular Insights into Gastrointestinal Dysfunction in Myotonic Dystrophy Types 1 & 2

**DOI:** 10.3390/ijms232314779

**Published:** 2022-11-26

**Authors:** Janel A. M. Peterson, Thomas A. Cooper

**Affiliations:** 1 Department of Molecular & Human Genetics, Baylor College of Medicine, Houston, TX 77030, USA; 2 Baylor College of Medicine, Department of Pathology & Immunology, Baylor College of Medicine, Houston, TX 77030, USA; 3 Baylor College of Medicine, Department of Integrative Physiology, Baylor College of Medicine, Houston, TX 77030, USA; 4 Baylor College of Medicine, Department of Molecular & Cellular Biology, Baylor College of Medicine, Houston, TX 77030, USA

**Keywords:** myotonic dystrophy type 1 (DM1), myotonic dystrophy type 2 (DM2), gastrointestinal dysfunction, smooth muscle, alternative splicing

## Abstract

Myotonic dystrophy (DM) is a highly variable, multisystemic disorder that clinically affects one in 8000 individuals. While research has predominantly focused on the symptoms and pathological mechanisms affecting striated muscle and brain, DM patient surveys have identified a high prevalence for gastrointestinal (GI) symptoms amongst affected individuals. Clinical studies have identified chronic and progressive dysfunction of the esophagus, stomach, liver and gallbladder, small and large intestine, and rectum and anal sphincters. Despite the high incidence of GI dysmotility in DM, little is known regarding the pathological mechanisms leading to GI dysfunction. In this review, we summarize results from clinical and molecular analyses of GI dysfunction in both genetic forms of DM, DM type 1 (DM1) and DM type 2 (DM2). Based on current knowledge of DM primary pathological mechanisms in other affected tissues and GI tissue studies, we suggest that misregulation of alternative splicing in smooth muscle resulting from the dysregulation of RNA binding proteins muscleblind-like and CUGBP-elav-like is likely to contribute to GI dysfunction in DM. We propose that a combinatorial approach using clinical and molecular analysis of DM GI tissues and model organisms that recapitulate DM GI manifestations will provide important insight into defects impacting DM GI motility.

## 1. Introduction

Myotonic dystrophy (DM) is an autosomal dominant neuromuscular disease and the most common adult-onset muscular dystrophy, clinically affecting one in 8000 individuals [1,2,3]. DM exists in two genetically distinct forms: DM type 1 (DM1, OMIM 160900) and DM type 2 (DM2, OMIM 602668). While both types are primarily characterized by myotonia and progressive muscle weakness, affected individuals experience highly variable multisystemic symptoms affecting the brain, heart, skeletal muscle, reproductive system, and gastrointestinal (GI) tract [4].

The genetic cause of DM1 was identified in 1992 as a CTG trinucleotide repeat expansion in the 3′ untranslated region of *dystrophia myotonica protein kinase* (*DMPK)* gene [5,6,7]. In affected individuals, the expanded repeats are 50 to > 4000 copies compared to 38 or fewer in healthy individuals. In 2001, the genetic etiology of DM2 was identified as a CCTG tetranucleotide repeat expansion in intron 1 of *CCHC-type zinc finger nucleic acid binding protein (CNBP)* gene, previously known as *ZNF9* [8]. Upon transcription of the expanded *DMPK* or *CNBP* allele, RNA with expanded CUG or CCUG repeats form double-stranded RNA hairpins that accumulate in nuclear foci. These expanded RNA aggregates result in a toxic gain of function consistent with dominant inheritance by binding and sequestering the muscleblind-like (MBNL) family of RNA binding proteins causing their loss of function [9,10,11]. Sequestration of MBNL reduces MBNL activity and dysregulates MBNL RNA targets. Other RNA binding proteins that are normally downregulated in adult tissues are upregulated in DM1 including CUGBP-elav-like (CELF) and hnRNPA1 [12,13,14]. CELF and MBNL RNA binding proteins regulate several aspects of RNA processing for hundreds of genes during development, including alternative splicing, polyadenylation, mRNA stability, and localization [11,15,16]. MBNL sequestration and CELF upregulation by expanded RNA dysregulates RNA processing globally, primarily leading to the incompatible expression of fetal protein isoforms in adult tissues, causing DM1 and DM2 features [9,11,17,18,19,20].

**Figure 1:**
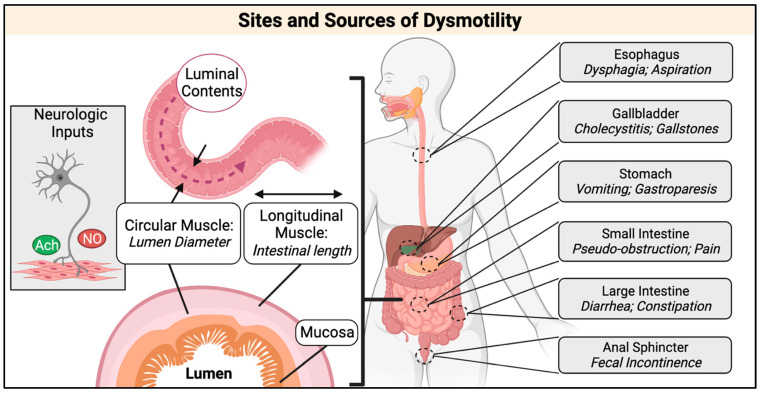
Sites and sources of GI dysmotility. **Left:** The smooth muscle layer is made up of inner circular and outer longitudinal muscle layers. When circular muscle contracts, the lumen diameter decreases; when it relaxes, it increases in diameter. Contraction of the longitudinal muscle decreases the length of the GI tract, while relaxation lengthens it. Coordinated contractions of the circular and longitudinal layers results in peristalsis, which propels luminal contents forward. Peristalsis is induced by inputs from the autonomic and enteric nervous systems. Acetylcholine (Ach) is the major excitatory neurotransmitter that induces contractions. Nitric oxide (NO) is the major inhibitory neurotransmitter that induces muscle relaxation. **Right:** GI symptoms are observed throughout the GI tract of DM affected individuals and affect movement of luminal contents. Created with BioRender.com.

Clinical and molecular analysis of DM has primarily focused on skeletal muscle, heart and brain pathologies. Clinical studies evaluating DM GI symptoms were largely conducted between the 1960s and early 2000s and demonstrated that affected individuals experience GI dysfunction in any region of the GI tract (Figure 1) [21,22,23,24,25,26]. More recent findings from DM symptom surveys brought attention to the high prevalence and significant burden of GI tract disturbances on the daily life and well-being within the DM community. As DM1 mortality rates are greater than DM2, studies investigating the pathogenic mechanisms of disease have predominantly focused on DM1 over DM2 despite clear GI dysmotility symptoms in both forms of the disease. 

GI tract function is regulated by neurogenic and myogenic factors and proper gut motility is essential for the consumption and digestion of food, uptake of water and nutrients, and excretion of waste. The coordinated contraction of smooth muscle lining the GI tract is necessary to grind, mix, and propel luminal contents forward (Figure 1). Disruption of the autonomic nervous system (ANS) or enteric nervous system (ENS), which induce smooth muscle contractions, or disruption of the contractile network of smooth muscle cells (SMCs) can impact GI tract function, leading to aspiration of a food bolus into the lungs, buildup of digesting material in the gut lumen, dehydration, fecal incontinence, and more [27]. Severe dysfunction not only disrupts daily life but also impacts mental and emotional health [22]. Clinical studies have established that those affected by DM can experience a range of symptoms throughout the entire GI tract; however, there is a lack of mechanistic understanding behind the observed dysfunction. This review provides an overview of current clinical understanding of DM1 and DM2 GI dysfunction and the cellular and molecular abnormalities that relate to potential mechanisms of disease. It is important to note that, as DM1 and DM2 were not distinguished from one another clinically until 1994 and genetically in 2001, studies prior to or between 1994 and 2001 may include both DM1 and DM2 individuals, both of which experience GI dysfunction. Throughout this review, studies conducted before the genetic discernment of DM1 and DM2 will refer to subjects as having “DM”. 

## 2. Clinical Findings

DM1 and DM2 are highly variable multisystemic diseases and the number of affected organ systems and their level of symptom severity widely differ between affected individuals, even within the same family [28]. Clinical studies have shown dysfunction across GI regions including the esophagus, stomach, small and large intestine, liver and gallbladder, and anal sphincter. One region may be more prominently affected than another within an individual, but the basis for variability between individuals and why different GI regions are affected within an individual is unknown. Identification of affected GI regions may also be complicated by the fact that symptoms of GI dysmotility, such as nausea, bloating, abdominal pain, and vomiting, are relatively non-specific. A recently published systematic review of DM1 genitourinary and lower GI conditions in clinical practice outlined 75 DM studies, their findings, and pitfalls; while the authors underline a lack of study standardization and small sample sizes throughout past DM1 GI clinical work, the information gathered from these studies confirms the existence of consistent GI symptoms in DM-affected individuals [29]. Regardless of the affected region, GI dysfunction negatively impacts one’s daily function and comfort, and in severe cases can be lethal [30,31,32]. Without proper movement of luminal contents, there are additional concerns for malabsorption and nutritional inadequacy [33,34], and small intestinal bacterial overgrowth (SIBO) [35], and postoperative complications due to anesthesia such as aspiration pneumonia [36]. Clear descriptions of DM GI dysfunction and improved understanding of the pathological mechanisms underlying DM GI symptoms will greatly improve diagnosis and symptom management. 

### 2.1. Prevalence 

Given the non-specific nature of GI symptoms, understanding the prevalence and type of GI symptoms experienced in DM1 and DM2 was essential to establishing an association between disease and GI dysmotility. One early study interviewed 40 DM affected individuals and 40 healthy controls and found that abdominal pain, difficulty swallowing, vomiting, chronic or episodic diarrhea, and anal incontinence occur more frequently in DM [37]. A patient-reported impact of symptoms in myotonic dystrophy type 1 (PRISM-1) study supported the common incidence of GI symptoms amongst DM-affected individuals, with 83% of 278 adult DM1 subjects having consistently experienced GI symptoms during the course of their disease [21]. A retrospective study of 913 DM1 affected and 180 DM2 affected individuals further maintained previous findings, with 79% of DM1 and 77% of DM2 patients reporting a history of at least one GI symptom [24]. Of those who completed an annual update form over the next five years in this study, a percentage of individuals who had previously reported no history of GI symptoms indicated the development of GI manifestations, such as trouble swallowing (27% DM1, 25% DM2) and constipation (14% DM1, 34% DM2), indicating progression of disease [24]. 

Within adult DM1 cohorts, trouble swallowing (48-55%), acid reflux (38-39%), and constipation (33-46%) are the most common GI symptoms experienced [23,24,32]. Only a handful of surveys have included or focused on DM2 affected individuals; of these surveys, trouble swallowing (29-52%) abdominal pain (62%) and constipation (53-62%) are the most common GI symptoms [24,25,38,39]. In one DM2-focused survey, 45% (13 of 29) of affected individuals indicated their GI symptoms to be the most disabling aspect of the disease [25].

Several variables affect the severity of GI symptoms in DM, which have been explored more in-depth in DM1 specifically. The prevalence of GI manifestations differs between the sexes, with DM1 affected females reported to experience significantly more GI symptoms than affected males [23]. Age of disease onset also affects symptom severity. Surveys of families affected by congenital or pediatric-onset DM1 have shown that GI symptoms are more prevalent and profound in this population, affecting 75% of those interviewed [40]. Severe dysfunction can lead to gastric tube feedings to cope with dysphagia and colostomy to manage fecal incontinence [40,41]. 

As GI function is influenced by environmental factors such as diet, exercise, pharmaceuticals, and an individual’s microbiome, issues with GI function may not always be attributed to DM or brought to a physician’s attention unless symptoms are severe and/or chronic. The embarrassment surrounding GI issues and bowel control have also been suspected to preclude conversations between patients and clinicians [42]. This indicates that current prevalence studies may underrepresent the true number and severity of affected individuals. Increasing the number of studies on GI dysfunction may aid the discussion of DM GI symptoms and help build confidence in affected individuals to evaluate and report their symptoms to their primary physician, potentially leading to specialized gastroenterological care.

### 2.2. Esophagus

**Figure 2:**
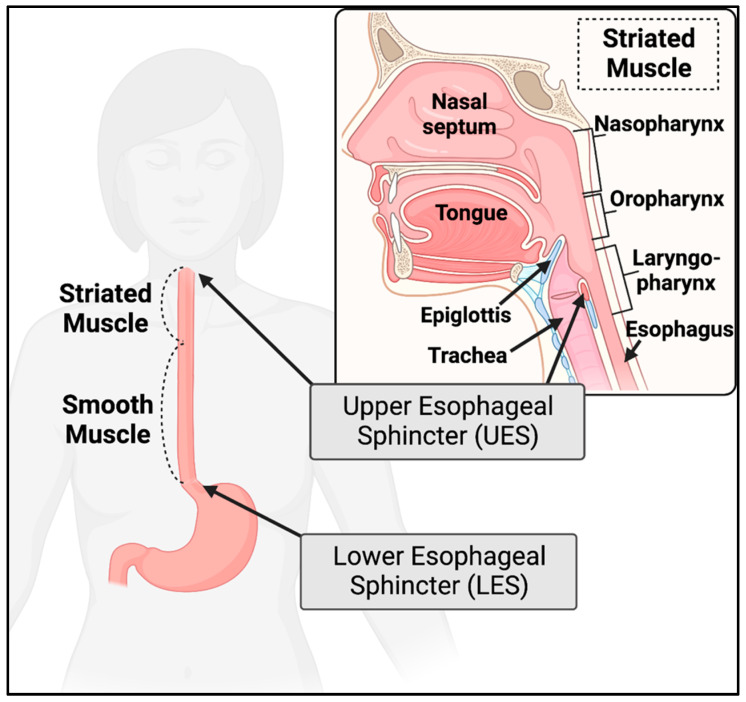
The mouth and proximal esophagus are predominantly striated muscle, while the distal esophagus is predominantly smooth muscle. The upper esophageal sphincter (UES) must relax to allow a food bolus to enter the esophagus. The lower esophageal sphincter (LES) must relax to allow luminal contents to enter the stomach. Created with BioRender.com.

One of the most common self-reported and clinically studied GI symptoms in DM is difficulty swallowing, known as dysphagia. A large concern of dysphagia is the increased risk for aspiration leading to pneumonia, pulmonary disease, and subsequent acute respiratory failure [30,36,43]. The high frequency of dysphagia in DM is most likely due to dysfunction of both striated muscle (tongue, proximal/cervical esophagus, upper esophageal sphincter) and smooth muscle (distal/thoracic esophagus, lower esophageal sphincter) during the swallowing phases (Figure 2) [44]. Dysfunction of both muscle types can affect swallowing and proper movement of a food bolus to the stomach. 

Changes in esophageal function can be evaluated by barium swallow tests, which, as barium is radio-opaque and can be seen on x-ray, visualizes esophageal movement during swallowing. DM-affected individuals often retain barium-containing liquid or solid meals in the oropharyngeal recesses, upper esophagus, and lower esophagus during barium swallow tests, with a tendency for significant delays in esophageal emptying time regardless of meal type [45,46,47,48]. In some cases, luminal contents have been observed flowing back into the pharynx after passing through the upper esophageal sphincter, fluid has run from the nose of affected individuals after swallowing, or swallowing required tilting the head backwards [45,46,49,50,51]. As barium swallow tests also provide radiographic evidence of reduced muscle contractions, barium was observed to passively “trickle” down the back of the tongue through the pharynx, the nasal passages did not close off and a considerable amount of barium remained in the recesses of the pharynx of a DM affected subject after swallowing [51]. Often, the esophagus is found to be dilated in DM cases compared to control subjects [45,48,50,52,53]. Given that the pharynx and proximal esophagus are dominated by striated muscle, which is known to have intrinsic dysfunction in DM [13,54,55,56,57], it is not surprising to find dysfunction of proximal esophageal mechanics.

In addition to barium swallows, other aspects of esophageal function can be measured by esophageal manometry, in which a probe is inserted into the esophagus to record changes in esophageal pressure at rest or during swallows. Clinical studies have consistently demonstrated that intraluminal pharyngoesophageal pressure of DM-affected individuals is reduced during swallowing compared to controls across studies [43,46,48,49,58,59]. Peristaltic contractions were often recorded as weak or absent in the pharynx and both upper and lower esophageal regions of DM-affected subjects; if contractions were present, the normal peristaltic contraction sequence was maintained similarly to controls [43,45,46,49,58,60,61,62]. Findings from barium and manometry assays have demonstrated that both striated and smooth muscle function of the esophagus is altered in DM subjects and that there is considerable variability in severity of esophageal dysfunction between DM affected individuals. 

The esophageal regions with particularly variable dysfunction are the upper and lower esophageal sphincters. The upper esophageal sphincter (UES) consists of skeletal muscle and is the junction between the pharynx and esophagus, which acts to prevent air from entering the esophagus during breathing or aspiration of esophageal contents during reflux into the pharynx and trachea [44]. When swallowing is initiated, the UES relaxes and allows the bolus to pass into the esophagus. Evaluation of the UES in DM has consistently shown reduced resting pressure and contraction amplitudes upon swallowing [43,46,48]. Likewise, the lower esophageal sphincter (LES) consists of smooth muscle and is the junction between the esophagus and stomach, preventing reflux of highly acidic gastric contents that can damage esophageal tissues [44]. Studies of the LES have shown diminished pressure and contraction amplitudes in DM affected subjects [46,48]. Delayed passage of luminal contents at the LES has been observed [50]. This may be due to prolonged duration of LES contraction [43,62]; however, some studies have been unable to reproduce these findings [43] or have identified increased duration of LES relaxation when compared to controls [59]. Defects in LES contraction would explain the high prevalence of heartburn or gastroesophageal reflux disease in DM affected individuals [48]. Variance in findings may be associated with disease duration and selection of study participants; while some studies selected DM-affected individuals with general GI complaints, others chose participants with severe GI manifestations, which are more likely to have more severe and measurable sphincter dysfunction. 

Most esophageal studies were performed prior to DM2 being identified. However, Tieleman et al. completed a dysphagia study on eight individuals with genetically confirmed DM2. Seven of the eight individuals (88%) exhibited dysphagia and retainment of solid food (88%), milk (75%), and/or saliva (25%) in the esophagus after swallow challenges [38]. Barium-containing milk leaked into the larynx of two subjects (25%) while solid food was detected in the larynx of one (13%) [38]. 

While only some individuals affected by DM1 may subjectively note difficulties in swallowing, each esophageal study reviewed has identified distinct abnormalities in upper and/or lower esophageal pressure and contraction in DM affected individuals with or without self-reported dysphagic symptoms [43,45,46,48,58,59]. This suggests that individuals adapt to and compensate for the muscular changes that occur with swallowing over time. Despite this, affected individuals are still at greater risk for aspiration pneumonitis and may be unaware of their propensity for esophageal dysfunction, which may lead to other serious secondary symptoms such as lower esophageal blockage [62,63]. 

While detecting physiological dysfunction is important, perception of esophageal symptoms can cause emotional distress, impact the social function of eating, and affect outcomes of clinical interventions. Pilz et al. conducted a swallow-related quality of life measurement (SWAL-QOL) in 75 DM1 individuals with dysphagia and 25 healthy controls. Individuals were asked swallow-related questions regarding general burden, food selection, eating duration, eating desire, fear of eating, communication, social functioning, and mental health, as well as two generic quality of life questions regarding fatigue and sleep. Symptom scoring was based on a scale of 0 for extreme impairment to 100 for no impairment. The overall SWAL-QOL score was significantly lower in DM1 affected individuals compared to controls and identified eating duration and food selection as top swallowing-related impairments [32]. Both impairments may result from oropharyngeal muscle weakness that prevents effective clearing of the mouth and throat of food, leading to more swallows during a meal and selection of foods easier to eat. This scoring system may allow physicians to develop a rehabilitation plan that can improve quality of life for those in the DM community experiencing dysphagia.

### 2.3. Stomach

Prior to larger clinical studies, individual case reports noted gastric complications in DM, such as a solid mass of indigestible material that caused gastric blockage [64] or little to no gastric peristalsis [52,60]. In a case of congenital DM, a newborn had difficulties with feeding, and while radiographic examination at eight weeks of age showed normal esophageal motility, the patient had delays in gastric emptying with 97% of the isotope remaining in the stomach after one hour [65]. Clinical studies have consistently identified significant defects in gastric emptying of individuals affected by DM1 compared to healthy age- and sex-matched control groups by radioisotope, ^13^C-acetate breath test, and ultrasound methods [47,66,67,68]. 

In radioisotope studies, subjects are provided a standardized, radiolabeled test meal that must be eaten within an allotted amount of time (i.e., 10 minutes). Using this method, DM1 affected individuals were shown to have a significant increase in time from end of meal ingestion until the stomach began emptying (gastric lag phase, when 10% of meal has emptied), slower overall emptying rate (percentage decrease gastric radioactivity per minute), and increased time for 50% of total radioisotope to empty from the stomach (gastric half-emptying time) [66]. Gastric emptying can also be measured using ^13^C-acetate breath tests [69]. After the consumption, absorption, and metabolism of a liquid test meal containing ^13^C-sodium acetate, levels of ^13^CO_2_ in the breath can be quantified over time and used to calculate half-emptying time [68,69]. ^13^C-acetate breath tests confirmed longer half-emptying times in DM1 affected individuals compared to controls, regardless of self-reported gastric symptoms [68]. Changes to gastric structures in addition to emptying can be quantitated by ultrasound. DM1 affected individuals were found to have a significantly greater antral area of the stomach between 120–300 min after meal ingestion and a significantly higher final emptying time compared to controls, regardless of their gastric symptom history [67]. 

No clinical studies have evaluated gastric function in DM2, however, symptom surveys that include DM2 affected individuals indicate that gastric involvement is likely [24].

### 2.4. Liver & Gallbladder

Once luminal contents are emptied from the stomach into the small intestine, bile, which is produced by the liver and stored and released by the gallbladder, is secreted to aid in the digestion of fats [70]. Smooth muscle contractions are necessary to release and move bile from the gallbladder into the common bile duct [70]; dysmotility of bile can contribute to the formation of gallstones and gallbladder inflammation (cholecystitis), often necessitating gallbladder removal [70]. Historically, cholecystitis and gallstones were commonly reported in DM affected individuals [71,72] and recent DM surveys have confirmed the high incidence of gallbladder issues, reporting 16.5% (151 of 913) of DM1 and 12.8% (23 of 180) of DM2 patients having had their gallbladder removed [24]. DM-associated gallbladder dysfunction has been measured by X-ray, where the gallbladders of nine DM-affected individuals were intravenously filled with radio-opaque dye and the gallbladder imaged over time after administration of cholecystokinin (CCK), a hormone that stimulates the release of bile into the intestine [49,70]. While all healthy controls emptied bile from the gallbladder within seven minutes after CCK administration, three of the nine DM patients had abnormal bile release, with one patient’s gallbladder emptying slowly and two patients not responding to CCK at all [49].

In addition to gallbladder dysfunction, abnormal liver enzyme levels have been consistently reported in DM affected individuals. The liver plays an integral role in digestion by generating bile and processing blood containing nutrients absorbed from the small intestine; dysfunction or disease of the liver will increase liver enzyme levels in serum. Table 1 lists the liver enzymes found to be elevated in DM affected individuals and the significance of these findings [23,73,74,75]. Rönnemaa et al. first reported a fivefold increase in gamma-glutamyl transferase (GGT) levels in 11 of 19 DM subjects, and these findings were supported in two more studies where 51% (27 of 53) and 65% (40 of 61) of DM subjects had abnormally elevated GGT levels, on average 1.5 times above the normal upper limit [23,73]. Increased GGT and alkaline phosphatase (ALP) levels often indicate decreased bile flow, known as cholestasis; given this, it is speculated that in DM, liver enzymes are elevated secondary to gallbladder and bile duct dysmotility, which would impair bile excretion and affect liver function [73]. Upon ultrasound investigation, abnormal retention of fat in the liver, known as steatosis, was observed in 34.4% (21 of 61) of DM1 patients, however, this finding may be a result from insulin resistance experienced by DM patients due to defects in insulin receptor expression [23]. 

### 2.5. Small & Large Intestine 

As previously noted, the non-specific symptoms of severe abdominal pain and distention, nausea and vomiting, constipation and/or diarrhea experienced by DM affected individuals are common and can indicate intestinal involvement in the disease. Often, the combination of these symptoms would suggest an intestinal blockage, however, early case studies of DM-affected individuals experiencing chronic diarrhea found no physical obstructions in the small or large intestine by X-ray examination [45,52,60,65,76,77,78,79,80,81]. Having signs and symptoms of mechanical obstruction without a physical blockage is known as pseudo-obstruction, which often occurs in the small or large intestine and results from GI dysmotility. 

Studies focused on small intestine motility of DM-affected individuals used manometry to measure contraction amplitude and duration. DM-affected individuals often had a dilated small bowel, low amplitude contractions, and altered contraction duration compared to unaffected controls [60,82]. While contraction force varied between affected and unaffected participants, the frequency of contractions remained the same, demonstrating that the peristaltic contraction pattern remained intact. This finding suggests that while contractile signals are present, the electrical stimulus and/or contractile response is disrupted, interfering with motor activity.

Similar to the small intestine, the large intestine of DM affected individuals is often markedly dilated, has low amplitude or, in severe cases, no peristaltic contractions, and a loss of segmentation (also known as haustration) that normally helps mix luminal contents and increases time for absorption of fluid and electrolytes [52,60,77,78,79,80,83,84]. In a small DM2 study, radiological assessment of colon transit time in 18 DM2 individuals identified delays in transit time in 24% of participants [25]. Pseudo-obstruction of the large intestine can cause constipation and contribute to fecal impaction. Depending on the severity of the impaction, treatment may initially involve restricting oral intake, intravenous fluids, and enemas [78]. Treatment can become increasingly invasive when medication and enemas fail to stimulate bowel movements and may result in a total colectomy [80]. 

Diseases that affect the small and large intestine are often associated with increased intestinal permeability, normally due to inflammation and impairment of the gut mucosa and intestinal barrier [85]. Early assessments of intestinal permeability in DM affected individuals by urine samples or blood tests revealed no consistent changes from controls [49,86]. Updated methods to assess intestinal permeability use metabolically inert markers that only transfer across the epithelium by passing through the intercellular space between cells (paracellular transport) and are subsequently excreted in the urine, such as chromium-51 labeled ethylenediamine tetraacetic acid (51CrEDTA) [85]. Since 51CrEDTA has a high molecular weight, only 1–3% of orally ingested 51CrEDTA will be excreted in the urine of a healthy individual; values greater than 3% indicate increased absorption across the intestinal tract [23,87]. An intestinal permeability assay using 51CrEDTA identified altered intestinal permeability (> 3% 51CrEDTA) in 52 out of 57 DM1-affected subjects (91.22%) [23], indicating that the intestinal barrier is impaired. Intestinal dysmotility can lead to inflammation and SIBO that can impact the intestinal barrier, and while detailed descriptions of DM intestinal biopsies are lacking, samples have been remarked as having “mild, chronic inflammatory changes” [49,52,59,77,81,84]. Steatosis and nonalcoholic fatty liver disease (NAFLD) may also contribute to altered gut permeability [88,89]. Further detailed examination of histological specimens and the development of NAFLD in DM would be needed to draw conclusions as to whether altered gut permeability is a primary or secondary symptom in DM and may be best studied in an animal model of the disease.

### 2.6. Rectum & Anal Sphincters

**Figure 3:**
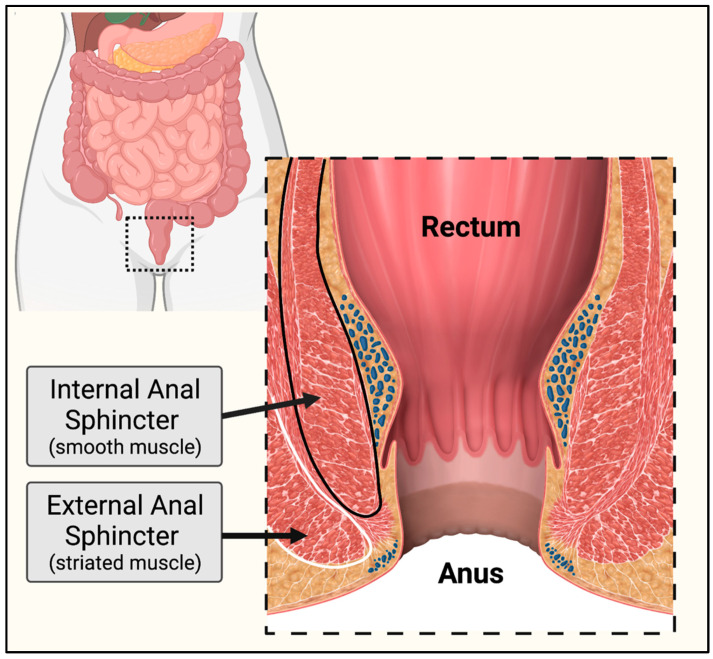
Cross section of rectum and anus. Fecal continence is dependent on the contraction and relaxation of the anal sphincter rings. The external anal sphincter (outlined in white) consists of striated muscle. The internal anal sphincter (outlined in black) arises from the muscularis of the large intestine and consists of smooth muscle. Normally, both sphincters will be contracted. Upon rectal distention by stool, the internal anal sphincter will involuntarily relax. Excretion of stool is controlled by voluntary relaxation of the external anal sphincter. Created with BioRender.com.

The rectum and anal sphincters play essential roles in controlling bowel movements. The rectum serves as a holding space for stool. The internal and external anal sphincters, which consist of smooth and striated muscle, respectively, control the release of stool (Figure 3). In one survey, 104 out of 152 (68%) of individuals affected by DM1 reported experiencing issues with bowel control, known as fecal incontinence. Of these 104 subjects, 38 (37%) reported changing their lifestyle as a result of their incontinence [26,37]. In DM1-affected children, severe constipation and fecal incontinence are highly prevalent and identified as the most debilitating symptoms of the disease, often affecting a child’s ability to be potty-trained [28,41].

Anorectal studies were performed in DM1 affected individuals and measured the amplitude, duration, and relaxation of sphincter contraction. Normally, the internal anal sphincter will involuntarily relax in response to distention of the rectum due to feces, known as the rectoanal inhibitory reflex (RAIR). The external anal sphincter provides voluntary control over the excretion of stool. In early studies, DM-affected subjects demonstrated abnormal RAIR and both the internal and external sphincters were slow to relax [49,90,91]. In a follow-up study including 13 DM-affected individuals, RAIR was induced by injecting 10–50 mL of air into the rectum and the maximum amplitude of relaxation, time to the maximum relaxation, time to return to resting pressure, and total duration of the reflex was recorded [59]. The maximum amplitude of relaxation was significantly lower than controls as previously observed and the duration of RAIR was significantly shorter in DM individuals as well, due to the shorter contraction phase after relaxation [59]. Surprisingly, anorectal studies have consistently observed tonic contractions of high amplitude following an occurrence of RAIR in all DM subjects but not healthy controls [59,90,91,92]. In severely affected cases, RAIR could not be induced during manometry and is suspected to be due to atrophy of the internal and external anal sphincters [28,80,90,92]. As fecal incontinence can be extremely distressing, permanent colostomy may be a final treatment decision should other methods fail to restore continence [41,92].

To our knowledge, no DM2 specific studies have been conducted to evaluate anorectal function or determine prevalence of fecal incontinence, and one survey that included 180 DM2-affected individuals reported <3 individuals with fecal incontinence [24]. As fecal incontinence can be a debilitating symptom that is difficult to discuss, empathetic doctor-patient conversations can alleviate feelings of isolation and allow for productive discussions focused on therapeutic intervention [42]. 

### 2.7. Enteric Nervous System

Control of GI smooth muscle motility is autonomous and predominantly controlled by the ENS. Acetylcholine (Ach) is the major excitatory neurotransmitter, while nitric oxide (NO) is the major inhibitory neurotransmitter [93]. Signals from the ENS are further regulated by interstitial cells of Cajal (ICCs) and platelet-derived growth factor receptor-expressing (PDGFR+) cells, which mediate excitatory or inhibitory signals reaching smooth muscle cells [93]. Coordinated electrical signals are necessary for coordinated contraction of longitudinal and circular smooth muscle, which allows for proper mixing and propulsion of luminal contents through the GI tract. Disruption of the ENS and its delivery of coordinated electrical signals, for example, through loss or degeneration of enteric nerves, will affect gut motility by altering the contractile properties of smooth muscle cells, leading to gastroparesis, chronic intestinal pseudo-obstruction, and constipation [94]. 

NO is predominately responsible for calcium (Ca^2+^) sensitization of the contractile apparatus, inhibition of Ca^2+^ channels, and activation of potassium channels within smooth muscle cells [95]. It has been suspected that changes in NO may be responsible for aspects of DM pathology given that NO receptors are expressed in smooth muscle cells and histological abnormities are rarely detected in smooth muscle of DM affected individuals [96]. Other studies extrapolate that abnormal contraction amplitudes and coordination in esophageal and anorectal motility could be explained by altered NO expression [59].

In addition to the ENS, sympathetic and parasympathetic input of the ANS also affects GI motility [94,97]. Overall autonomic nervous system function was assessed in DM patients in 1985 and 1990 and showed normal vasomotor function and circulatory reflex responses, all indicating that there are no signs of sympathetic neuropathy in evaluated DM subjects [98,99]. These findings suggest that defects in intrinsic neuronal control by the ENS and/or SMCs are contributing to GI dysmotility rather than defects in extrinsic pathways. Given the small sample sizes of these studies (*n* = 8 and *n* = 30), further evaluation of the ANS and its impact on GI muscle function would be informative, especially in an animal model of the disease that would reduce additional environmental and genetic variables.

Pharmacological agents that increase Ach release (i.e., metoclopramide and cisapride) have been used to increase GI motility in DM patients experiencing GI delay but have had mixed success in treating symptoms [47,66]. While these medications may increase the amount of neurotransmitter secreted to normal levels if there are fewer neurons, it may alternatively increase the amount of neurotransmitter present to activate more receptors found on SMCs, should receptor activity or expression be disrupted by DM [100]. Other pharmacological agents that act similarly to the gut hormone motilin have also been used to increase GI motility, such as erythromycin. Motilin receptors are present on enteric nerves and SMCs and when bound by their agonist stimulate muscle contractions by increasing cytosolic levels of Ca^2+^ [101]. Clinical case studies that used erythromycin also had variable outcomes regarding GI symptom management [66]. A lack of consistent pharmacological response further supports that GI disease heterogeneously manifests in DM, affecting both the ENS and SMCs and resulting in GI dysmotility.

While the majority of histological evaluations of affected tissues have not shown significant morphological changes other than mild inflammatory changes in GI mucosa [52,59,81], a handful of case studies have identified alterations in the myenteric nerve plexus or atrophy of smooth muscle [28,77]. For instance, transmission electron microscopy of a colonic sample from a DM affected male experiencing megacolon showed a >75% loss in neuron number, but with otherwise normal myenteric plexus structure [77]. Difficulties lie in determining primary versus secondary histological pathologies, as the impact of chronic inflammation on smooth muscle and nerve function is unclear. However, a reduction in enteric neurons would impact muscle function, contribute to muscle atrophy, and result in GI dysmotility, as well.

### 2.8. Clinical Conclusions

Given that GI function is naturally variable between individuals and is influenced by a number of environmental factors such as diet, exercise, pharmaceuticals, and an individual’s microbiome, GI dysfunction in DM might not receive attention unless symptoms are severe and/or chronic. However, clear and consistent clinical data exists supporting GI dysfunction as a pervasive symptom in DM but with little known regarding the mechanisms of dysmotility. We focused on the GI regions with the greatest evidence of dysfunction, however, GI mucosa and gut hormone secretion influence motility and may contribute to motility deficits [93,94]. More non-invasive studies could be conducted that focus on gut transit time in DM-affected individuals to gain a broader sense of altered motility throughout disease progression. Such large-scale motility studies have been accomplished through the integration of blue dyed food and a mobile phone application to log ingestion and excretion times [102]. Given the difficulty or invasive nature of clinical testing and the natural GI variability between individuals, systematic testing of model organisms that recapitulate DM GI symptoms may offer the clearest path in identifying defects of specific GI tissue types and regions. 

## 3. Molecular Findings

The pathophysiological mechanisms that lead to DM GI dysfunction are not understood at the cellular or molecular levels; however, there are insights from GI tissue studies and from information that can be extrapolated from mechanisms discovered in other DM-affected tissues, such as skeletal muscle, heart, and brain. Given the heterogenous nature of GI tissue, the interdependence of the nervous system and smooth muscle in development and function [103,104], as well as the expression of *DMPK* and *CNBP* in both nervous and smooth muscle cell types, it is unlikely that pathogenic changes will only in occur in either the ENS or SMCs. While both neurogenic and myogenic factors would contribute to the observed GI symptoms, the lack of clinical evidence for ANS and ENS dysfunction in DM-associated GI dysmotility in combination with the overall higher expression of *DMPK* and *CNBP* in SMCs (Genotype-Tissue Expression Portal [GTEx]) suggests that prominent GI phenotypes most likely arise from some aspect of smooth muscle dysfunction. Intrinsic striated muscle dysfunction is thoroughly described throughout the DM field, further supporting that inherent defects of the contractile apparatus or electrophysiological features of muscle cells will lead to dysfunction and DM symptomology. Given the current state of knowledge, this review will primarily focus on the potential molecular changes in smooth muscle that would lead to chronic GI dysfunction in DM.

### 3.1. DMPK and CNBP Expression in the Gastrointestinal Tract Smooth Muscle

The potential for pathogenicity in the GI tract in DM1 or DM2 relies on the expression of expanded *DMPK* or *CNBP* RNA, respectively. *DMPK* RNA is known to be expressed throughout the GI tract in mice and humans [105,106]. Ribonuclear aggregates of expanded *DMPK* RNA have been detected in DM1 affected gallbladder smooth muscle [107] but, to our knowledge, has yet to be investigated in other GI tissues. Similarly, *CNBP* RNA aggregates have been detected in DM2 affected vascular smooth muscle but has yet to be assessed in GI smooth muscle [108]. This expression pattern indicates that SMCs can express expanded *DMPK* or *CNBP* alleles. As expanded *DMPK* and *CNBP* RNA will aggregate in the nucleus and not undergo translation, protein expression is suspected to be reduced in tissues of affected individuals leading to haploinsufficiency [109,110,111,112] or will undergo repeat-associated non-AUG (RAN) translation, resulting in the expression of toxic, repeat expansion proteins that interfere with cellular processes [113,114,115]. While only a few studies have investigated the impact of RAN translation protein products expressed from CUG or CCUG repeat RNA on cellular function, which primarily focused on brain [116,117], animal models have been used more extensively to evaluate the effects of overall DMPK or CNBP protein loss

A *DMPK* knockout mouse model was used to explore *DMPK* haploinsufficiency in DM1 pathogenesis and did not recapitulate DM1 symptoms of the heart or skeletal muscle [118]. However, another study showed that *DMPK* loss in cardiomyocytes impacts Ca^2+^ cycling by decreasing Ca^2+^ uptake by the sarcoplasmic reticulum [106]. In SMCs, DMPK protein has been shown to localize at neuromuscular junctions and SMC dense bodies of human tissues and to play a role in phosphorylating myosin light-chain phosphatase, which is necessary to induce smooth muscle relaxation [106,119,120]. How a reduction in DMPK protein levels would interfere with SMC function is unknown. Interestingly, one study has connected the transcriptional regulator serum response factor (SRF) to DMPK expression, where depletion of SRF in GI smooth muscle decreased DMPK levels; however, decoupling the effects of SRF loss versus DMPK loss on GI function in this study is difficult [121]. As research has focused on the impact of DMPK loss in striated muscle, of which it has little to no impact on striated muscle function, the role of DMPK in the GI tract has not been fully elucidated.

Recent studies have identified potential roles for *CNBP* haploinsufficiency in DM2 pathogenesis. CNBP KO mouse models recapitulate aspects of DM2, including muscle atrophy and multisystem abnormalities [112,122]. Heterozygous loss of CNBP in mice led to severe muscle wasting at an advanced age, while mice with homozygous loss of CNBP developed muscle atrophy early in life [112,122]. *Drosophila* deficient of CNBP (dCNBP knockdown) have also shown age-dependent locomotor defects that are fully rescued with overexpression of *Drosophila* or human CNBP [123]. CNBP deficient flies were found to have impaired polyamine metabolism, recapitulating polyamine deficiency observed in human DM2 skeletal muscle samples and fly locomotor activity was rescued with polyamine supplementation [123]. The role of polyamine metabolism in the GI tract, however, has not been fully explored. Despite these animal model findings, conflicting evidence still exists regarding whether CNBP expression is truly reduced in human DM2 tissues [112,124,125].

Although the impact of *DMPK* or *CNBP* haploinsufficiency on GI function remains underexplored, the combination of expanded *DMPK* or *CNBP* RNA expression, RAN translation, and reduction of DMPK or CNBP protein levels in GI tissues contribute to the complexity of DM pathogenic mechanisms and GI dysfunction. While DMPK or CNBP protein loss and RAN translation protein products most likely affect disease onset and severity, the expression of expanded CUG or CCUG repeat RNA is known to greatly interfere with RNA binding protein expression and negatively impact RNA processing. The pathogenic changes caused by dysregulated RNA processing have consistently been demonstrated in striated muscle in both cell and animal models, providing significant support for this pathogenic mechanism in DM smooth muscle.

### 3.2. The Potential Roles of Disrupted MBNL and CELF Functions in Smooth Muscle RNA Processing and Regulation

**Figure 4:**
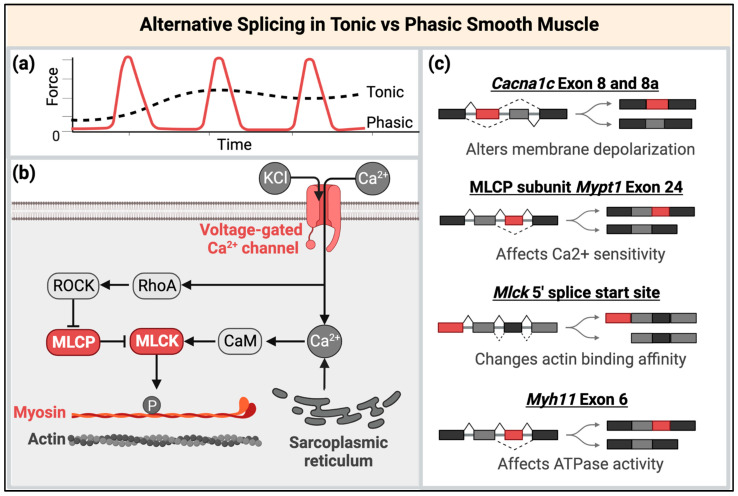
Alternative splicing controls aspects of tonic or phasic SMC types. (**a**) Tonic smooth muscle maintains contraction over time, while phasic smooth muscle undergoes strong contractions followed by relaxation. (**b**) Increased cytoplasmic levels of calcium (Ca^2+^) induce contractions in SMCs. Proteins in red have alternative splicing patterns that are associated with determining tonic or phasic smooth muscle type. (**c**) Specific alternative splicing events alter protein function in smooth muscle. Splicing of voltage-gated ion channels, such as L-Type Ca^2+^ Channel CaV1.2, encoded by *Cacna1c*, varies between smooth muscle types. Mutually exclusive splicing of exon 8/8a of *Cacna1c* affects membrane depolarization and sensitivity of smooth muscle types to drug treatments [134,135]. Exon 24 (31 nt) of the myosin phosphatase regulatory subunit *Mypt1* (or *Ppp1r12a*) is typically included in phasic smooth muscle postnatally [136]. Inclusion of exon 24 impacts Ca^2+^ sensitivity by decreasing sensitivity to cGMP, which in turn increases the force of contraction [137]. Isoforms of myosin light chain kinase (MLCK) have altered protein binding affinities for actin filaments; alternative 5′ splice start sites generate short and long MLCK isoforms that co-exist throughout development, but the shortening of the N-terminus alters the affinity for MLCK for actin-containing filaments [138]. The ratios of these two isoforms varies between phasic or tonic smooth muscle. Splicing in of a 21 nt exon near the motor domain of smooth muscle myosin heavy chain (*Myh11*) increases ATPase activity of MYH11, which is coupled with an increase in velocity of actin filament movement in vitro [139,140]. This exon is predominantly included in phasic smooth muscle that has quicker contractions compared to tonic smooth muscle. Created with BioRender.com.

While multiple types of RNA processing are known to be disrupted in DM, the best characterized is misregulated alternative splicing. Pre-mRNA alternative splicing is essential for development and tissue-specific functions, as slight variations of existing proteins are needed to fulfill specific cellular mechanisms. With approximately 20,000 genes in the human genome, alternative splicing allows expression of different protein isoforms from a single gene based on cellular needs. In smooth muscle, studies have begun to associate alternative splicing changes with smooth muscle’s ability to (1) remain phenotypically plastic, allowing for dedifferentiation from a contractile phenotype to a proliferative one, and (2) vary in cell type depending on functional need, such as producing tonic (slow) contractile cells that maintain contractions over time or phasic (fast) contractile cells that undergo strong contractions followed by relaxation (Figure 4a) [126,127]. Bioinformatic assessment of transcript splicing from mouse exon junction arrays identified 4244 alternative splicing events between proliferative and differentiated aortic (tonic) smooth muscle and 5193 alternative splicing events between proliferative and differentiated bladder (phasic) smooth muscle, suggesting that alternative splicing plays a key role in determining and/or maintaining SMC state and type [128]. Alternative splicing of mRNA encoding contractile network proteins, such as alpha-tropomyosin (*Tpm1)*, alpha-actinin-1 (*Actn1)*, myocardin (*Myocd)*, and vinculin (*Vcl)*, have been shown to switch during differentiation, thus altering SMC differentiation state and contractile ability [128,129,130,131]. These specific splicing events occur during SMC differentiation across SMC cell types. Splicing perturbation of single RNAs encoding contractile network proteins have demonstrated functional and phenotypic changes as well. For instance, altered splicing of the calmodulin binding protein caldesmon (CaD) affects Ca^2+^ sensitivity and thus muscle contraction patterns. Splicing of an alternative “spacer” of CaD, exons 3b and 4, creates an isoform (denoted H-CaD) that is specific to differentiated smooth muscle and larger than the isoform primarily expressed in striated muscle [129]. Inclusion of this spacer in H-CaD modulates cross-bridge cycling of myosin heads [132]. When H-CaD, but not the smaller CaD isoform, was knocked out in a zebrafish model, propulsive peristalsis was enhanced [133]. Research regarding the mechanisms defining tonic versus phasic SMCs have also gained traction. Alternative splicing patterns in several transcripts have been identified that have strong associations with tonic or phasic SMC types and have altered SMC contraction force. The ability for SMCs to switch between differentiated and proliferative cell states as well as tonic or phasic cell types based on isoform expression patterns has potential implications in disease, which are outlined in Figure 4b,c.

Expression of expanded CUG or CCUG repeat RNA interferes with alternative splicing patterns by creating high affinity binding sites for the MBNL protein family and upregulating expression of the CELF protein family [9,10,141,142]. As MBNL and CELF are developmentally regulated proteins, the sequestration and loss of activity of MBNL or upregulation of CELF ultimately dysregulates RNA splicing, switching from adult to fetal protein expression patterns that do not support adult tissue functions [9,11,17,18,19,20]. The pathogenicity of this switch in protein isoform expression is well defined in DM affected striated muscle. For instance, in DM1-affected skeletal muscle, mis-splicing of chloride ion channel *CLCN1* causes myotonia [55,143] and misregulated splicing of Ca^2+^ pump *CACNA1S* and insulin receptor *INSR* transcripts likely contribute to muscle weakness and insulin resistance, respectively [56,144]. Aspects of DM1 heart conduction deficits have been attributed to mis-splicing of the sodium channel encoded by *SCN5A* [145,146]. As in striated muscle, MBNL and CELF proteins are expressed in smooth muscle tissue throughout development and in adulthood, giving potential for RNA processing dysregulation upon expression of CUG or CCUG repeat RNA [16,147,148,149]. Despite this, little is known regarding the specific role of MBNL and CELF in maintaining alternative splicing patterns during smooth muscle development and GI tract function.

MBNL protein has been observed to colocalize with *DMPK* or *CNBP* nuclear RNA aggregates in DM1 and DM2 smooth muscle samples [107,108]. While MBNL colocalization in the nucleus suggests that MBNL activity could be decreased, loss of MBNL function was not confirmed in either study. MBNL has been demonstrated to co-regulate the repression of exon 3 of *Tpm1* in smooth muscle; typically, exon 3 is included in transcripts expressed in skeletal muscle, heart and brain, but not smooth muscle [150]. MBNL was also found to promote inclusion of exon 14 in *Mypt1* (*Ppp1r12a*) transcripts in mouse *Mbnl* KO skeletal muscle and heart samples [151]. Given the integral role of the MYPT1 subunit of myosin light-chain phosphatase in regulating Ca^2+^ sensitivity and inducing SMC relaxation, the inclusion of this 171 nt exon may affect phosphatase function; however, the effect of this exon in striated or smooth muscle function is unexplored. Examination of MBNL loss of function in smooth muscle has begun in a germline zebrafish mbnl KO model that demonstrated altered alternative splicing events in multiple tissues, including the GI system, however, phenotyping assays have yet to be conducted to assess GI motility [152].

Antagonistically to MBNL, CELF proteins are upregulated in DM1 [142] and potentially DM2 [153,154,155]. The CELF protein family has been shown to regulate *Actn1* mRNA by controlling the inclusion of a smooth-muscle specific exon and exclusion of non-muscle specific exon [156]. Inclusion of the non-muscle associated exon generates a protein containing a Ca^2+^-binding EF hand domain; the smooth muscle predominant exon generates a nonfunctional domain that alters Ca^2+^ sensitivity [157]. CELF proteins can also affect RNA turnover. For instance, tyrosine phosphorylation of CELF2 in vascular smooth muscle increases the association of CELF2 with *COX2/PTGS2* mRNA, which in turn increases mRNA stability [158]. Knowledge regarding the role of the CELF protein family in smooth muscle is greatly lacking, but CELF upregulation has been demonstrated to disrupt many aspects of skeletal and cardiac muscle function in cell and animal models that recapitulate aspects of DM disease pathology [16,141,159,160].

### 3.3. Molecular Conclusions

Taken together, there is a strong indication that misregulated alternative splicing events in smooth muscle contribute to GI dysfunction in DM. The use of model organisms, such as mouse and zebrafish, combined with human clinical studies and sample analysis will help identify conserved mechanisms of pathogenesis. Understanding which events have the strongest effects on GI function will provide key targets for therapeutic interventions, help guide clinicians in medication and surgical procedure selections, and prevent the use of invasive procedures that disrupt daily life. A clearer understanding of alternative splicing regulation in GI tract function will also contribute greatly to our knowledge of GI development and provide necessary background for identifying the genetic etiology of other sources of GI dysmotility.

## Figures and Tables

**Table 1:** Liver enzymes found to be elevated in DM.

Serum Enzyme (Abbreviation)	Significance of Elevated Levels
Alkaline phosphatase (ALP)	Damage or disease of liver or bone; cholestasis
Alanine aminotransferase (ALT or GPT)	Hepatocellular damage
Gamma-glutamyl transferase (GGT)	Disease or damage of liver or bile ducts; cholestasis
5′ nucleotidase	Cholestasis, hepatitis, ischemia, or tumor

## Data Availability

Not applicable.

## References

[1] Yum K., Wang E.T., Kalsotra A. (2017). Myotonic Dystrophy: Disease Repeat Range, Penetrance, Age of Onset, and Relationship between Repeat Size and Phenotypes. Curr. Opin. Genet. Dev..

[2] Suominen T., Bachinski L.L., Auvinen S., Hackman P., Baggerly K.A., Angelini C., Peltonen L., Krahe R., Udd B. (2011). Population Frequency of Myotonic Dystrophy: Higher than Expected Frequency of Myotonic Dystrophy Type 2 (DM2) Mutation in Finland. Eur. J. Hum. Genet..

[3] Johnson N.E., Butterfield R.J., Mayne K., Newcomb T., Imburgia C., Dunn D., Duval B., Feldkamp M.L., Weiss R.B. (2021). Population Based Prevalence of Myotonic Dystrophy Type 1 Using Genetic Analysis of State-Wide Blood Screening Program. Neurology.

[4] Kumar A., Agarwal S., Agarwal D., Phadke S.R. (2013). Myotonic Dystrophy Type 1 (DM1): A Triplet Repeat Expansion Disorder. Gene.

[5] Brook J.D., McCurrach M.E., Harley H.G., Buckler A.J., Church D., Aburatani H., Hunter K., Stanton V.P., Thirion J.-P., Hudson T. (1992). Molecular Basis of Myotonic Dystrophy: Expansion of a Trinucleotide (CTG) Repeat at the 3′ End of a Transcript Encoding a Protein Kinase Family Member. Cell.

[6] Mahadevan M., Tsilfidis C., Sabourin L., Shutler G., Amemiya C., Jansen G., Neville C., Narang M., Barceló J., O’Hoy K. (1992). Myotonic Dystrophy Mutation: An Unstable CTG Repeat in the 3′ Untranslated Region of the Gene. Science.

[7] Pizzuti A., Friedman D.L., Caskey C.T. (1993). The Myotonic Dystrophy Gene. Arch. Neurol..

[8] Liquori C.L., Ricker K., Moseley M.L., Jacobsen J.F., Kress W., Naylor S.L., Day J.W., Ranum L.P.W. (2001). Myotonic Dystrophy Type 2 Caused by a CCTG Expansion in Intron 1 of ZNF9. Science.

[9] Miller J.W., Urbinati C.R., Teng-umnuay P., Stenberg M.G., Byrne B.J., Thornton C.A., Swanson M.S. (2000). Recruitment of Human Muscleblind Proteins to (CUG)n Expansions Associated with Myotonic Dystrophy. EMBO J..

[10] Fardaei M., Rogers M.T., Thorpe H.M., Larkin K., Hamshere M.G., Harper P.S., Brook J.D. (2002). Three Proteins, MBNL, MBLL and MBXL, Co-Localize in Vivo with Nuclear Foci of Expanded-Repeat Transcripts in DM1 and DM2 Cells. Hum. Mol. Genet..

[11] Jiang H., Mankodi A., Swanson M.S., Moxley R.T., Thornton C.A. (2004). Myotonic Dystrophy Type 1 Is Associated with Nuclear Foci of Mutant RNA, Sequestration of Muscleblind Proteins and Deregulated Alternative Splicing in Neurons. Hum. Mol. Genet..

[12] Li M., Zhuang Y., Batra R., Thomas J.D., Li M., Nutter C.A., Scotti M.M., Carter H.A., Wang Z.J., Huang X.-S. (2020). HNRNPA1-Induced Spliceopathy in a Transgenic Mouse Model of Myotonic Dystrophy. Proc. Natl. Acad. Sci. USA.

[13] Lee K., Li M., Manchanda M., Batra R., Charizanis K., Mohan A., Warren S.A., Chamberlain C.M., Finn D., Hong H. (2013). Compound Loss of Muscleblind-like Function in Myotonic Dystrophy. EMBO Mol. Med..

[14] Paul S., Dansithong W., Kim D., Rossi J., Webster N.J., Comai L., Reddy S. (2006). Interaction of Musleblind, CUG-BP1 and HnRNP H Proteins in DM1-associated Aberrant IR Splicing. EMBO J..

[15] Wang E.T., Cody N.A.L., Jog S., Biancolella M., Wang T.T., Treacy D.J., Luo S., Schroth G.P., Housman D.E., Reddy S. (2012). Transcriptome-Wide Regulation of Pre-MRNA Splicing and MRNA Localization by Muscleblind Proteins. Cell.

[16] Ladd A.N., Charlet-B. N., Cooper T.A. (2001). The CELF Family of RNA Binding Proteins Is Implicated in Cell-Specific and Developmentally Regulated Alternative Splicing. Mol. Cell. Biol..

[17] Taneja K.L., McCurrach M., Schalling M., Housman D., Singer R.H. (1995). Foci of Trinucleotide Repeat Transcripts in Nuclei of Myotonic Dystrophy Cells and Tissues. J. Cell Biol..

[18] Mankodi A., Urbinati C.R., Yuan Q.-P., Moxley R.T., Sansone V., Krym M., Henderson D., Schalling M., Swanson M.S., Thornton C.A. (2001). Muscleblind Localizes to Nuclear Foci of Aberrant RNA in Myotonic Dystrophy Types 1 and 2. Hum. Mol. Genet..

[19] André L.M., van Cruchten R.T.P., Willemse M., Wansink D.G. (2019). (CTG)n Repeat-Mediated Dysregulation of MBNL1 and MBNL2 Expression during Myogenesis in DM1 Occurs Already at the Myoblast Stage. PLoS ONE.

[20] Lin X., Miller J.W., Mankodi A., Kanadia R.N., Yuan Y., Moxley R.T., Swanson M.S., Thornton C.A. (2006). Failure of MBNL1-Dependent Post-Natal Splicing Transitions in Myotonic Dystrophy. Hum. Mol. Genet..

[21] Heatwole C., Bode R., Johnson N., Quinn C., Martens W., McDermott M.P., Rothrock N., Thornton C., Vickrey B., Victorson D. (2012). Patient-Reported Impact of Symptoms in Myotonic Dystrophy Type 1 (PRISM-1). Neurology.

[22] Peric S., Heatwole C., Durovic E., Kacar A., Nikolic A., Basta I., Marjanovic A., Stevic Z., Lavrnic D., Stojanovic V.R. (2017). Prospective Measurement of Quality of Life in Myotonic Dystrophy Type 1. Acta Neurol. Scand..

[23] Perna A., Maccora D., Rossi S., Nicoletti T.F., Zocco M.A., Riso V., Modoni A., Petrucci A., Valenza V., Grieco A. (2020). High Prevalence and Gender-Related Differences of Gastrointestinal Manifestations in a Cohort of DM1 Patients: A Perspective, Cross-Sectional Study. Front. Neurol..

[24] Hilbert J.E., Barohn R.J., Clemens P.R., Luebbe E.A., Martens W.B., McDermott M.P., Parkhill A.L., Tawil R., Thornton C.A., Moxley R.T. (2017). High Frequency of Gastrointestinal Manifestations in Myotonic Dystrophy Type 1 and Type 2. Neurology.

[25] Tieleman A.A., van Vliet J., Jansen J.B.M.J., van der Kooi A.J., Borm G.F., van Engelen B.G.M. (2008). Gastrointestinal Involvement Is Frequent in Myotonic Dystrophy Type 2. Neuromuscul. Disord..

[26] Petty R.K.H., Eugenicos M.P., Hamilton M.J., Farrugia M.E., Robb Y., Ballantyne R., Gregory H., McWilliam C., Longman C. (2019). The Prevalence of Faecal Incontinence in Myotonic Dystrophy Type 1. Neuromuscul. Disord..

[27] Cheng L.K., O’Grady G., Du P., Egbuji J.U., Windsor J.A., Pullan A.J. (2010). Gastrointestinal System. Wiley Interdiscip. Rev. Syst. Biol. Med..

[28] Degraeuwe J., Laecke E.V., Muynck M.D., Biervliet S.V., Velde S.V., Winckel M.V. (2011). Faecal Incontinence Due to Atrophy of the Anal Sphincter in Myotonic Dystrophy: A Case Report. Acta Gastro-Enterol. Belg..

[29] Fisette-Paulhus I., Gagnon C., Girard-Côté L., Morin M. (2022). Genitourinary and Lower Gastrointestinal Conditions in Patients with Myotonic Dystrophy Type 1: A Systematic Review of Evidence and Implications for Clinical Practice. Neuromuscul. Disord..

[30] Mathieu J., Allard P., Potvin L., Prévost C., Bégin P. (1999). A 10-Year Study of Mortality in a Cohort of Patients with Myotonic Dystrophy. Neurology.

[31] Gagnon C., Chouinard M.C., Laberge L., Veillette S., Bégin P., Breton R., Jean S., Brisson D., Gaudet D., Mathieu J. (2010). Health Supervision and Anticipatory Guidance in Adult Myotonic Dystrophy Type 1. Neuromuscul. Disord..

[32] Pilz W., Passos V.L., Verdonschot R.J., Meijers J., Roodenburg N., Halmans Y., Faber C.G., Kremer B., Baijens L.W.J. (2020). Swallow-Related Quality of Life and Oropharyngeal Dysphagia in Myotonic Dystrophy. Eur. Arch. Oto-Rhino-Laryngol..

[33] Motlagh B., MacDonald J.R., Tarnopolsky M.A. (2005). Nutritional Inadequacy in Adults with Muscular Dystrophy. Muscle Nerve.

[34] Malik H.Z., Sharma G., Moreno C., Parcha S.P. (2022). A Medley of Malnutrition and Myotonic Dystrophy: Twice Unlucky. Cureus.

[35] Tarnopolsky M.A., Pearce E., Matteliano A., James C., Armstrong D. (2010). Bacterial Overgrowth Syndrome in Myotonic Muscular Dystrophy Is Potentially Treatable. Muscle Nerve.

[36] Ishizawa Y., Yamaguchi H., Dohi S., Koyama K. (1986). A Serious Complication Due to Gastrointestinal Malfunction in a Patient with Myotonic Dystrophy. Anesth. Analg..

[37] Rönnblom A., Forsberg H., Danielsson A. (1996). Gastrointestinal Symptoms in Myotonic Dystrophy. Scand. J. Gastroenterol..

[38] Tieleman A.A., Knuijt S., van Vliet J., de Swart B.J.M., Ensink R., Engelen B.G.M. (2009). van Dysphagia Is Present but Mild in Myotonic Dystrophy Type 2. Neuromuscul. Disord..

[39] Wenninger S., Stahl K., Montagnese F., Schoser B. (2020). Utility and Results from a Patient-Reported Online Survey in Myotonic Dystrophies Types 1 and 2. Eur. Neurol..

[40] Hunter M., Ekstrom A., Campbell C., Hung M., Bounsanga J., Bates K., Adams H.R., Luebbe E., Moxley R.T., Heatwole C. (2019). Patient-reported Study of the Impact of Pediatric-onset Myotonic Dystrophy. Muscle Nerve.

[41] Kerr T.P., Robb S.A., Clayden G.S. (2002). Lower Gastrointestinal Tract Disturbance in Congenital Myotonic Dystrophy. Eur. J. Pediatr..

[42] Whitehead W.E. (2005). Diagnosing and Managing Fecal Incontinence: If You Don’t Ask, They Won’t Tell. Gastroenterology.

[43] Garrett J.M., DuBose T.D., Jackson J.E., Norman J.R. (1969). Esophageal and Pulmonary Disturbances in Myotonia Dystrophica. Arch. Intern. Med..

[44] Goyal R.K., Chaudhury A. (2008). Physiology of Normal Esophageal Motility. J. Clin. Gastroenterol..

[45] Pruzanski W., Profis A. (1966). Dysfunction of the Alimentary Tract in Myotonic Dystrophy. Isr. J. Med Sci..

[46] Schuman B.M., Rinaldo J.A., Darnley J.D. (1965). Visceral Changes in Myotonic Dystrophy. Ann. Intern. Med..

[47] Horowitz M., Maddox A., Maddern G.J., Wishart J., Collins P.J., Shearman D.J.C. (1987). Gastric and Esophageal Emptying in Dystrophia Myotonica Effect of Metoclopramide. Gastroenterology.

[48] Costantini M., Zaninotto G., Anselmino M., Marcon M., Iurilli V., Boccù C., Feltrin G.P., Angelini C., Ancona E. (1996). Esophageal Motor Function in Patients with Myotonic Dystrophy. Am. J. Dig. Dis..

[49] Harvey J.C., Sherbourne D.H., Siegel C.I. (1965). Smooth Muscle Involvement in Myotonic Dystrophy. Am. J. Med..

[50] Hughes D.T.D., Swann J.C., Gleeson J.A., Lee F.I. (1965). Abnormalities In Swallowing Associated With Dystrophia Myotonica. Brain.

[51] Kramer P., Atkinson M., Wyman S.M., Ingelfinger F.J. (1957). The Dynamics of Swallowing. II. Neuromuscular Dysphagia of Pharynx. J. Clin. Investig..

[52] Goldberg H.I., Sheft D.J. (1972). Esophageal and Colon Changes in Myotonia Dystrophica. Gastroenterology.

[53] Nowak T.V., Ionasescu V., Anuras S. (1982). Gastrointestinal Manifestations of the Muscular Dystrophies. Gastroenterology.

[54] André L.M., Ausems C.R.M., Wansink D.G., Wieringa B. (2018). Abnormalities in Skeletal Muscle Myogenesis, Growth, and Regeneration in Myotonic Dystrophy. Front. Neurol..

[55] Mankodi A., Takahashi M.P., Jiang H., Beck C.L., Bowers W.J., Moxley R.T., Cannon S.C., Thornton C.A. (2002). Expanded CUG Repeats Trigger Aberrant Splicing of ClC-1 Chloride Channel Pre-MRNA and Hyperexcitability of Skeletal Muscle in Myotonic Dystrophy. Mol. Cell.

[56] Tang Z.Z., Yarotskyy V., Wei L., Sobczak K., Nakamori M., Eichinger K., Moxley R.T., Dirksen R.T., Thornton C.A. (2011). Muscle Weakness in Myotonic Dystrophy Associated with Misregulated Splicing and Altered Gating of CaV1.1 Calcium Channel. Hum. Mol. Genet..

[57] Bachinski L.L., Baggerly K.A., Neubauer V.L., Nixon T.J., Raheem O., Sirito M., Unruh A.K., Zhang J., Nagarajan L., Timchenko L.T. (2014). Most Expression and Splicing Changes in Myotonic Dystrophy Type 1 and Type 2 Skeletal Muscle Are Shared with Other Muscular Dystrophies. Neuromuscul. Disord..

[58] Swick H.M., Werlin S.L., Dodds W.J., Hogan W.J. (1981). Pharyngoesophageal Motor Function in Patients with Myotonic Dystrophy. Ann. Neurol..

[59] Lecointe-Besancon I., Leroy F., Devroede G., Chevrollier M., Lebeurier F., Congard P., Arhan P. (1999). A Comparative Study of Esophageal and Anorectal Motility in Myotonic Dystrophy. Dig. Dis. Sci..

[60] Lewis T.D., Daniel E.E. (1981). Gastroduodenal Motility in a Case of Dystrophia Myotonica. Gastroenterology.

[61] Ghazaleh S., Nehme C., Khader Y., Hasan S., Nawras A. (2020). Combined Achalasia and Cricopharyngeal Achalasia in a Patient with Type 1 Myotonic Dystrophy: A Case Report. Gastroenterol. Hepatol. Bed Bench.

[62] Sato H., Mizuno K., Hashimoto S., Takatsuna M., Terai S. (2020). Achalasia in a Patient with Myotonic Dystrophy. Intern. Med..

[63] Ogasawara N., Sato K., Tsutsumiuchi M., Kanzaki M., Uesaka Y. (2017). Steakhouse Syndrome in Myotonic Dystrophy. Intern. Med..

[64] Kuiper D.H. (1971). Gastric Bezoar in a Patient with Myotonic Dystrophy. Am. J. Dig. Dis..

[65] Bodensteiner J.B., Grunow J.E. (1984). Gastroparesis in Neonatal Myotonic Dystrophy. Muscle Nerve.

[66] Rönnblom A., Andersson S., Hellström P.M., Danielsson Å. (2002). Gastric Emptying in Myotonic Dystrophy. Eur. J. Clin. Investig..

[67] Bellini M., Alduini P., Costa F., Tosetti C., Pasquali L., Pucciani F., Tornar A., Mammini C., Siciliano G., Maltinti G. (2002). Gastric Emptying in Myotonic Dystrophic Patients. Dig. Liver Dis..

[68] Tanaka Y., Kato T., Nishida H., Yamada M., Koumura A., Sakurai T., Hayashi Y., Kimura A., Hozumi I., Araki H. (2013). Is There a Difference in Gastric Emptying between Myotonic Dystrophy Type 1 Patients with and without Gastrointestinal Symptoms?. J. Neurol..

[69] Braden B., Adams S., Duan L.-P., Orth K.-H., Maul F.-D., Lembcke B., Hör G., Caspary W.F. (1995). The [13C]Acetate Breath Test Accurately Reflects Gastric Emptying of Liquids in Both Liquid and Semisolid Test Meals. Gastroenterology.

[70] Housset C., Chrétien Y., Debray D., Chignard N. (2016). Functions of the Gallbladder. Compr. Physiol..

[71] Schwindt W.D., Bernhardt L.C., Peters H.A. (1969). Cholelithiasis and Associated Complications of Myotonia Dystrophica. Postgrad. Med..

[72] Chiu V.S.W. (1962). Gastrointestinal Disturbances in Myotonic Dystrophica. Aga Annu. Meet. Abstr..

[73] Achiron A., Barak Y., Magal N., Shohat M., Cohen M., Barar R., Gadoth N. (1998). Abnormal Liver Test Results in Myotonic Dystrophy. J. Clin. Gastroenterol..

[74] Rönnemaa T., Alaranta H., Viikari J., Tilvis R., Falck B. (1987). Increased Activity Of Serum Γ-Glutamyltransferase In Myotonic Dystrophy. Acta Med. Scand..

[75] Heatwole C.R., Miller J., Martens B., Moxley R.T. (2006). Laboratory Abnormalities in Ambulatory Patients With Myotonic Dystrophy Type 1. Arch. Neurol..

[76] Bruinenberg J., Rieu P., Gabreëls F., Tolboom J. (1996). Intestinal Pseudo-obstruction Syndrome in a Child with Myotonic Dystrophy. Acta Paediatr..

[77] Yoshida M.M., Krishnamurthy S., Wattchow D.A., Furness J.B., Schuffler M.D. (1988). Megacolon in Myotonic Dystrophy Caused by a Degenerative Neuropathy of the Myenteric Plexus. Gastroenterology.

[78] Brunner H.G., Hamel B.C., Rieu P., Höweler C.J., Peters F.T. (1992). Intestinal Pseudo-Obstruction in Myotonic Dystrophy. J. Med. Genet..

[79] Sartoretti C., Sartoretti S., DeLorenzi D., Buchmann P. (1996). Intestinal Non-Rotation and Pseudoobstruction in Myotonic Dystrophy: Case Report and Review of the Literature. Int. J. Color. Dis..

[80] Glaser A.M., Johnston J.H., Gleason W.A., Rhoads J.M. (2015). Myotonic Dystrophy as a Cause of Colonic Pseudoobstruction: Not Just Another Constipated Child. Clin. Case Rep..

[81] Pelizzo G., Calcaterra V., Villanacci V., Mura G.B., Bassotti G. (2018). Myotonic Dystrophy Type 1 and Pseudo-Obstruction in a Child with Smooth Muscle α-Actin Deficiency and Eosinophilic Myenteric Plexitis. Turk. J. Gastroenterol..

[82] Nowak T.V., Anuras S., Brown B.P., Ionasescu V., Green J.B. (1984). Small Intestinal Motility in Myotonic Dystrophy Patients. Gastroenterology.

[83] Cheng H.M., Mah K.K., Seluakumaran K. (2020). Defining Physiology: Principles, Themes, Concepts. Volume 2, Neurophysiology and Gastrointestinal Systems.

[84] Weiner M. (1978). Myotonic Megacolon in Myotonic Dystrophy. Am. J. Roentgenol..

[85] Vanuytsel T., Tack J., Farre R. (2021). The Role of Intestinal Permeability in Gastrointestinal Disorders and Current Methods of Evaluation. Front. Nutr..

[86] Sjaastad O. (1975). Intestinal Absorption in Myotonic Dystrophy. Acta Neurol. Scand..

[87] Bjarnason I., Macpherson A., Hollander D. (1995). Intestinal Permeability: An Overview. Gastroenterology.

[88] Shieh K., Gilchrist J.M., Promrat K. (2010). Frequency and Predictors of Nonalcoholic Fatty Liver Disease in Myotonic Dystrophy. Muscle Nerve.

[89] Miele L., Valenza V., Torre G.L., Montalto M., Cammarota G., Ricci R., Mascianà R., Forgione A., Gabrieli M.L., Perotti G. (2009). Increased Intestinal Permeability and Tight Junction Alterations in Nonalcoholic Fatty Liver Disease. Hepatology.

[90] Eckardt V.F., Nix W. (1991). The Anal Sphincter in Patients with Myotonic Muscular Dystrophy. Gastroenterology.

[91] Hamel-Roy J., Devroede G., Arhan P., Tetreault J., Lemieux B., Scott H. (1984). Functional Abnormalities of the Anal Sphincters in Patients with Myotonic Dystrophy. Gastroenterology.

[92] Abercrombie J.F., Rogers J., Swash M. (1998). Faecal Incontinence in Myotonic Dystrophy. J. Neurol. Neurosurg. Psychiatry.

[93] Sanders K.M., Koh S.D., Ro S., Ward S.M. (2012). Regulation of Gastrointestinal Motility—Insights from Smooth Muscle Biology. Nat. Rev. Gastroenterol..

[94] Camilleri M. (2021). Gastrointestinal Motility Disorders in Neurologic Disease. J. Clin. Investig..

[95] Sanders K.M., Ward S.M. (2019). Nitric Oxide and Its Role as a Non-adrenergic, Non-cholinergic Inhibitory Neurotransmitter in the Gastrointestinal Tract. Brit. J. Pharmacol..

[96] Bellini M., Biagi S., Stasi C., Costa F., Mumolo M.G., Ricchiuti A., Marchi S. (2006). Gastrointestinal Manifestations in Myotonic Muscular Dystrophy. World J. Gastroenterol..

[97] Browning K.N., Verheijden S., Boeckxstaens G.E. (2017). The Vagus Nerve in Appetite Regulation, Mood, and Intestinal Inflammation. Gastroenterology.

[98] Aminoff M.J., Beckley D.J., McIlroy M.B. (1985). Autonomic Function in Myotonic Dystrophy. Arch. Neurol..

[99] Olofsson B.-O., Niklasson U., Forsberg H., Bjerle P., Andersson S., Henriksson A. (1990). Assessment of Autonomic Nerve Function in Myotonic Dystrophy. J. Auton. Nerv. Syst..

[100] Fairhurst A.S., Lorenzen L., Reavie D. (1976). Altered Tracheal Smooth Muscle Activities in an Animal Model of Human Myotonic Dystrophy. Life Sci..

[101] Huang J., Zhou H., Mahavadi S., Sriwai W., Lyall V., Murthy K.S. (2005). Signaling Pathways Mediating Gastrointestinal Smooth Muscle Contraction and MLC20 Phosphorylation by Motilin Receptors. Am. J. Physiol. Liver Physiol..

[102] Asnicar F., Leeming E.R., Dimidi E., Mazidi M., Franks P.W., Khatib H.A., Valdes A.M., Davies R., Bakker E., Francis L. (2021). Blue Poo: Impact of Gut Transit Time on the Gut Microbiome Using a Novel Marker. Gut.

[103] Graham H.K., Maina I., Goldstein A.M., Nagy N. (2017). Intestinal Smooth Muscle Is Required for Patterning the Enteric Nervous System. J. Anat..

[104] Hao M.M., Foong J.P.P., Bornstein J.C., Li Z.L., Berghe P.V., Boesmans W. (2016). Enteric Nervous System Assembly: Functional Integration within the Developing Gut. Dev. Biol..

[105] Jansen G., Groenen P.J.T.A., Bächner D., Jap P.H.K., Coerwinkel M., Oerlemans F., van den Broek W., Gohlsch B., Pette D., Plomp J.J. (1996). Abnormal Myotonic Dystrophy Protein Kinase Levels Produce Only Mild Myopathy in Mice. Nat. Genet..

[106] Kaliman P., Catalucci D., Lam J.T., Kondo R., Gutiérrez J.C.P., Reddy S., Palacín M., Zorzano A., Chien K.R., Ruiz-Lozano P. (2005). Myotonic Dystrophy Protein Kinase Phosphorylates Phospholamban and Regulates Calcium Uptake in Cardiomyocyte Sarcoplasmic Reticulum*. J. Biol. Chem..

[107] Cardani R., Mancinelli E., Saino G., Bonavina L., Meola G. (2008). A Putative Role of Ribonuclear Inclusions and MBNL1 in the Impairment of Gallbladder Smooth Muscle Contractility with Cholelithiasis in Myotonic Dystrophy Type 1. Neuromuscul. Disord..

[108] Lukáš Z., Falk M., Feit J., Souček O., Falková I., Štefančíková L., Janoušová E., Fajkusová L., Zaorálková J., Hrabálková R. (2012). Sequestration of MBNL1 in Tissues of Patients with Myotonic Dystrophy Type 2. Neuromuscul. Disord..

[109] Maeda M., Taft C.S., Bush E.W., Holder E., Bailey W.M., Neville H., Perryman M.B., Bies R.D. (1995). Identification, Tissue-Specific Expression, and Subcellular Localization of the 80- and 71-KDa Forms of Myotonic Dystrophy Kinase Protein (∗). J. Biol. Chem..

[110] Furling D., Lam L.T., Agbulut O., Butler-Browne G.S., Morris G.E. (2003). Changes in Myotonic Dystrophy Protein Kinase Levels and Muscle Development in Congenital Myotonic Dystrophy. Am. J. Pathol..

[111] Salvatori S., Fanin M., Trevisan C.P., Furlan S., Reddy S., Nagy J.I., Angelini C. (2005). Decreased Expression of DMPK: Correlation with CTG Repeat Expansion and Fibre Type Composition in Myotonic Dystrophy Type 1. Neurol. Sci..

[112] Wei C., Stock L., Schneider-Gold C., Sommer C., Timchenko N.A., Timchenko L. (2018). Reduction of Cellular Nucleic Acid Binding Protein Encoded by a Myotonic Dystrophy Type 2 Gene Causes Muscle Atrophy. Mol. Cell. Biol..

[113] Zu T., Gibbens B., Doty N.S., Gomes-Pereira M., Huguet A., Stone M.D., Margolis J., Peterson M., Markowski T.W., Ingram M.A.C. (2011). Non-ATG–Initiated Translation Directed by Microsatellite Expansions. Proc. Natl. Acad. Sci. USA.

[114] Ash P.E.A., Bieniek K.F., Gendron T.F., Caulfield T., Lin W.-L., DeJesus-Hernandez M., van Blitterswijk M.M., Jansen-West K., Paul J.W., Rademakers R. (2013). Unconventional Translation of C9ORF72 GGGGCC Expansion Generates Insoluble Polypeptides Specific to C9FTD/ALS. Neuron.

[115] Mori K., Weng S.-M., Arzberger T., May S., Rentzsch K., Kremmer E., Schmid B., Kretzschmar H.A., Cruts M., Broeckhoven C.V. (2013). The C9orf72 GGGGCC Repeat Is Translated into Aggregating Dipeptide-Repeat Proteins in FTLD/ALS. Science.

[116] Zu T., Cleary J.D., Liu Y., Bañez-Coronel M., Bubenik J.L., Ayhan F., Ashizawa T., Xia G., Clark H.B., Yachnis A.T. (2017). RAN Translation Regulated by Muscleblind Proteins in Myotonic Dystrophy Type 2. Neuron.

[117] Banez-Coronel M., Ranum L.P.W. (2019). Repeat-Associated Non-AUG (RAN) Translation: Insights from Pathology. Lab. Investig..

[118] Carrell S.T., Carrell E.M., Auerbach D., Pandey S.K., Bennett C.F., Dirksen R.T., Thornton C.A. (2016). Dmpk Gene Deletion or Antisense Knockdown Does Not Compromise Cardiac or Skeletal Muscle Function in Mice. Hum. Mol. Genet..

[119] van der Ven P.F.M., Jansen G., van Kuppevelt T.H.M.S.M., Perryman M.B., Lupa M., Dunne P.W., ter Laak H.J., Jap P.H.K., Veerkamp J.H., Epstein H.F. (1993). Myotonic Dystrophy Kinase Is a Component of Neuromuscular Junctions. Hum. Mol. Genet..

[120] Murányi A., Zhang R., Liu F., Hirano K., Ito M., Epstein H.F., Hartshorne D.J. (2001). Myotonic Dystrophy Protein Kinase Phosphorylates the Myosin Phosphatase Targeting Subunit and Inhibits Myosin Phosphatase Activity. FEBS Lett..

[121] Lee M.Y., Park C., Ha S.E., Park P.J., Berent R.M., Jorgensen B.G., Corrigan R.D., Grainger N., Blair P.J., Slivano O.J. (2017). Serum Response Factor Regulates Smooth Muscle Contractility via Myotonic Dystrophy Protein Kinases and L-Type Calcium Channels. PLoS ONE.

[122] Chen W., Wang Y., Abe Y., Cheney L., Udd B., Li Y.-P. (2007). Haploinsuffciency for Znf9 in Znf9+/− Mice Is Associated with Multiorgan Abnormalities Resembling Myotonic Dystrophy. J. Mol. Biol..

[123] Coni S., Falconio F.A., Marzullo M., Munafò M., Zuliani B., Mosti F., Fatica A., Ianniello Z., Bordone R., Macone A. (2021). Translational Control of Polyamine Metabolism by CNBP Is Required for Drosophila Locomotor Function. eLife.

[124] Margolis J.M., Schoser B.G., Moseley M.L., Day J.W., Ranum L.P.W. (2006). DM2 Intronic Expansions: Evidence for CCUG Accumulation without Flanking Sequence or Effects on ZNF9 MRNA Processing or Protein Expression. Hum. Mol. Genet..

[125] Santoro M., Fontana L., Maiorca F., Centofanti F., Massa R., Silvestri G., Novelli G., Botta A. (2018). Expanded [CCTG]n Repetitions Are Not Associated with Abnormal Methylation at the CNBP Locus in Myotonic Dystrophy Type 2 (DM2) Patients. Biochim. et Biophys. Acta (BBA)-Mol. Basis Dis..

[126] Fisher S.A. (2010). Vascular Smooth Muscle Phenotypic Diversity and Function. Physiol. Genom..

[127] Nguyen A.T., Gomez D., Bell R.D., Campbell J.H., Clowes A.W., Gabbiani G., Giachelli C.M., Parmacek M.S., Raines E.W., Rusch N.J. (2013). Smooth Muscle Cell Plasticity. Circ. Res..

[128] Llorian M., Gooding C., Bellora N., Hallegger M., Buckroyd A., Wang X., Rajgor D., Kayikci M., Feltham J., Ule J. (2016). The Alternative Splicing Program of Differentiated Smooth Muscle Cells Involves Concerted Non-Productive Splicing of Post-Transcriptional Regulators. Nucleic Acids Res..

[129] Kashiwada K., Nishida W., Hayashi K., Ozawa K., Yamanaka Y., Saga H., Yamashita T., Tohyama M., Shimada S., Sato K. (1997). Coordinate Expression of α-Tropomyosin and Caldesmon Isoforms in Association with Phenotypic Modulation of Smooth Muscle Cells*. J. Biol. Chem..

[130] Nakagaki-Silva E.E., Gooding C., Llorian M., Jacob A.G., Richards F., Buckroyd A., Sinha S., Smith C.W. (2019). Identification of RBPMS as a Mammalian Smooth Muscle Master Splicing Regulator via Proximity of Its Gene with Super-Enhancers. eLife.

[131] Kanoldt V., Kluger C., Barz C., Schweizer A.-L., Ramanujam D., Windgasse L., Engelhardt S., Chrostek-Grashoff A., Grashoff C. (2020). Metavinculin Modulates Force Transduction in Cell Adhesion Sites. Nat. Commun..

[132] Guo H., Wang C.-L.A. (2005). Specific Disruption of Smooth Muscle Caldesmon Expression in Mice. Biochem. Biophys. Res. Commun..

[133] Abrams J., Davuluri G., Seiler C., Pack M. (2012). Smooth Muscle Caldesmon Modulates Peristalsis in the Wild Type and Non-innervated Zebrafish Intestine. Neurogastroenterol. Motil..

[134] Morinaga A., Ito J., Niimi T., Maturana A.D. (2019). RBM20 Regulates CaV1.2 Surface Expression by Promoting Exon 9* Inclusion of CACNA1C in Neonatal Rat Cardiomyocytes. Int. J. Mol. Sci..

[135] Hu Z., Liang M.C., Soong T.W. (2017). Alternative Splicing of L-Type CaV1.2 Calcium Channels: Implications in Cardiovascular Diseases. Genes.

[136] Khatri J.J., Joyce K.M., Brozovich F.V., Fisher S.A. (2001). Role of Myosin Phosphatase Isoforms in CGMP-Mediated Smooth Muscle Relaxation*. J. Biol. Chem..

[137] Zheng X., Reho J.J., Wirth B., Fisher S.A. (2015). TRA2β Controls Mypt1 Exon 24 Splicing in the Developmental Maturation of Mouse Mesenteric Artery Smooth Muscle. Am. J. Physiol. Physiol..

[138] Smith L., Parizi-Robinson M., Zhu M.-S., Zhi G., Fukui R., Kamm K.E., Stull J.T. (2002). Properties of Long Myosin Light Chain Kinase Binding to F-Actin in Vitro and in Vivo *. J. Biol. Chem..

[139] Rovner A.S., Freyzon Y., Trybus K.M. (1997). An Insert in the Motor Domain Determines the Functional Properties of Expressed Smooth Muscle Myosin Isoforms. J. Muscle Res. Cell Motil..

[140] Kelley C.A., Takahashi M., Yu J.H., Adelstein R.S. (1993). An Insert of Seven Amino Acids Confers Functional Differences between Smooth Muscle Myosins from the Intestines and Vasculature. J. Biol. Chem..

[141] Ho T.H., Bundman D., Armstrong D.L., Cooper T.A. (2005). Transgenic Mice Expressing CUG-BP1 Reproduce Splicing Mis-Regulation Observed in Myotonic Dystrophy. Hum. Mol. Genet..

[142] Kuyumcu-Martinez N.M., Wang G.-S., Cooper T.A. (2007). Increased Steady-State Levels of CUGBP1 in Myotonic Dystrophy 1 Are Due to PKC-Mediated Hyperphosphorylation. Mol. Cell.

[143] Charlet-B. N., Savkur R.S., Singh G., Philips A.V., Grice E.A., Cooper T.A. (2002). Loss of the Muscle-Specific Chloride Channel in Type 1 Myotonic Dystrophy Due to Misregulated Alternative Splicing. Mol. Cell.

[144] Savkur R.S., Philips A.V., Cooper T.A. (2001). Aberrant Regulation of Insulin Receptor Alternative Splicing Is Associated with Insulin Resistance in Myotonic Dystrophy. Nat. Genet..

[145] Pang P.D., Alsina K.M., Cao S., Koushik A.B., Wehrens X.H.T., Cooper T.A. (2018). CRISPR-Mediated Expression of the Fetal Scn5a Isoform in Adult Mice Causes Conduction Defects and Arrhythmias. J. Am. Heart Assoc..

[146] Freyermuth F., Rau F., Kokunai Y., Linke T., Sellier C., Nakamori M., Kino Y., Arandel L., Jollet A., Thibault C. (2016). Splicing Misregulation of SCN5A Contributes to Cardiac-Conduction Delay and Heart Arrhythmia in Myotonic Dystrophy. Nat. Commun..

[147] Brimacombe K.R., Ladd A.N. (2007). Cloning and Embryonic Expression Patterns of the Chicken CELF Family. Dev. Dyn..

[148] Kanadia R.N., Johnstone K.A., Mankodi A., Lungu C., Thornton C.A., Esson D., Timmers A.M., Hauswirth W.W., Swanson M.S. (2003). A Muscleblind Knockout Model for Myotonic Dystrophy. Science.

[149] Pascual M., Vicente M., Monferrer L., Artero R. (2006). The Muscleblind Family of Proteins: An Emerging Class of Regulators of Developmentally Programmed Alternative Splicing. Differentiation.

[150] Gooding C., Edge C., Lorenz M., Coelho M.B., Winters M., Kaminski C.F., Cherny D., Eperon I.C., Smith C.W.J. (2013). MBNL1 and PTB Cooperate to Repress Splicing of Tpm1 Exon 3. Nucleic Acids Res..

[151] Du H., Cline M.S., Osborne R.J., Tuttle D.L., Clark T.A., Donohue J.P., Hall M.P., Shiue L., Swanson M.S., Thornton C.A. (2010). Aberrant Alternative Splicing and Extracellular Matrix Gene Expression in Mouse Models of Myotonic Dystrophy. Nat. Struct. Mol. Biol..

[152] Hinman M.N., Richardson J.I., Sockol R.A., Aronson E.D., Stednitz S.J., Murray K.N., Berglund J.A., Guillemin K. (2021). Zebrafish Mbnl Mutants Model Physical and Molecular Phenotypes of Myotonic Dystrophy. Dis. Model. Mech..

[153] Pelletier R., Hamel F., Beaulieu D., Patry L., Haineault C., Tarnopolsky M., Schoser B., Puymirat J. (2009). Absence of a Differentiation Defect in Muscle Satellite Cells from DM2 Patients. Neurobiol. Dis..

[154] Sznajder Ł.J., Swanson M.S. (2019). Short Tandem Repeat Expansions and RNA-Mediated Pathogenesis in Myotonic Dystrophy. Int. J. Mol. Sci..

[155] Salisbury E., Schoser B., Schneider-Gold C., Wang G.-L., Huichalaf C., Jin B., Sirito M., Sarkar P., Krahe R., Timchenko N.A. (2009). Expression of RNA CCUG Repeats Dysregulates Translation and Degradation of Proteins in Myotonic Dystrophy 2 Patients. Am. J. Pathol..

[156] Gromak N., Matlin A.J., Cooper T.A., Smith C.W.J. (2003). Antagonistic Regulation of α-Actinin Alternative Splicing by CELF Proteins and Polypyrimidine Tract Binding Protein. RNA.

[157] Waites G.T., Graham I.R., Jackson P., Millake D.B., Patel B., Blanchard A.D., Weller P.A., Eperon I.C., Critchley D.R. (1992). Mutually Exclusive Splicing of Calcium-Binding Domain Exons in Chick Alpha-Actinin. J. Biol. Chem..

[158] Louis I.V.-S., Dickson A.M., Bohjanen P.R., Wilusz C.J. (2013). CELFish Ways to Modulate MRNA Decay. Biochim. et Biophys. Acta.

[159] Kalsotra A., Xiao X., Ward A.J., Castle J.C., Johnson J.M., Burge C.B., Cooper T.A. (2008). A Postnatal Switch of CELF and MBNL Proteins Reprograms Alternative Splicing in the Developing Heart. Proc. Natl. Acad. Sci. USA.

[160] Dhaenens C.M., Tran H., Frandemiche M.-L., Carpentier C., Schraen-Maschke S., Sistiaga A., Goicoechea M., Eddarkaoui S., Brussels E.V., Obriot H. (2011). Mis-Splicing of Tau Exon 10 in Myotonic Dystrophy Type 1 Is Reproduced by Overexpression of CELF2 but Not by MBNL1 Silencing. Biochim. et Biophys. Acta (BBA)-Mol. Basis Dis..

